# M. Fallot on Hydrocephalus

**Published:** 1822-03-01

**Authors:** 


### *Hydrocephalus.**

The celebrated Frank, of Vienna, has remarked, that "when hydrocephalus is curable the diagnosis is uncertain—but that when the diagnosis is certain, the case is hopeless." In this country, where the practice in acute diseases is far more efficient than on the Continent, there is every reason to believe that very many cases of hydrocephalus acutus (that is the cerebral excitement which ends in effusion) are annually cured. We do not indeed say, that after effusion, to any extent, has taken place in the brain, we can save the patient; but we can often prevent the effusion.

Dr. Fallot has circumstantially detailed a case, which, he thinks, was hydrocephalus successfully treated. The patient was a girl eight years of age, born of sound parents, but herself and two sisters having large heads and delicate constitutions. The girl, M. B. was exposed to severe cold on Christmas-day, 1820, and complained of head-ache, chilliness, and lassitude in the evening. For several days afterwards she continued dull, and complaining of pain about the occipital region. On the 1st of January, 1821, she vomited her food, and still continued unwell and irritable. On the 4th the vomiting returned, and being now worse than usual, Dr. Fallot was called in. He found the girl lying on her back, and unwilling to move from that position, having constant inclination to vomit, flushed face, intolerance of light, red dry tongue, burning skin, small, hard, quick pulse, lancinating pains in the head, especially over the orbits. Six leeches behind the ears—a semicupium. In the evening, there being little amelioration, six leeches were applied to the temples, a purgative lavement exhibited, and lemonade ordered for drink. 5th January. The night was passed without sleep, or with short and broken slumbers. A purplish eruption was now visible over the whole surface, without diminution of the fever, or gastric irritability. Six leeches to the epigastrium—gum water acidulated with tartaric acid. In the evening the head-ache, especially of the right orbit and temple, was more violent than ever, causing the child to utter piercing cries. Four leeches to the orbit; mustard pediluvia. 8th. The eruptive disease has run its stages, and desquammation of the cuticle, as in scarlatina, has commenced; but without any mitigation of the constitutional symptoms; the fever having a daily exacerbation or paroxysm at two o'clock, P. M. The patient complains continually of her head, to which she frequently applies her hand. Constipation is obstinate, the glysters producing no effect. Irritability excessive; the little patient not permitting either of her sisters to approach her bed. On the morning of the 9th our author found his patient altered much for the worse. The pulse was become so

---

* Journ. Complem. October 1821. Dr. Fallot.

quick and irregular that he could not count them—the face drawn—the eyes incapable of bearing the least degree of light—continual cries of "my head! my head!" even when apparently slumbering. Our author now, for the first time, suspected hydrocephalus acutus, and determined to act vigorously. He proposed a seton in the neck, which was refused. He therefore enveloped the feet in sinapisms, and prescribed a grain of calomel every three hours, changing the lemonade drink for an antimonial solution. The head was covered with ice, which was renewed as fast as it thawed.

12th. There being no sensible amendment yet, the seton was permitted, the girl evincing little sensibility to the pain. When she fell into a slumber of a few minutes, she awoke with a start, and crying out with the pain in her head. One grain of calomel and one of digitalis night and morning. An infusion of arnica montana—the antimonial drink continued.

15th. She this day opened the left eye, and with it viewed the objects around her bed, but kept her hand on the other eye. Had two mucous greenish stools to day—a cloud in the urine, which has become somewhat more abundant—the pulse a little more regular and better developed—the cries less piercing—no other change of any consequence. The child refused all medicine, and only took beer for drink.

The night between the 16th and 17th was a most severe one; the breathing being oppressed—the anxiety extreme—the patient attempting to raise herself, but wanting the power. When elevated by others, the head fell to one side or other. The heat of skin was burning and unequal—somnolency, interrupted by startings and crying out. About three o'clock in the morning, convulsive movements over the whole body. At day-light, horrible squinting, the right pupil being dilated, and scarcely sensible to a lighted candle. The intellects, however, appeared sound, when the patient was roused from her slumbers. The features were much altered, the skin burning, pulse small and irregular, grindings of the teeth, piercing cries. Cold solutions applied to the head instead of ice—mustard pediluvia—half a drachm of mercurial ointment to be rubbed on the inside of the thighs, and two grains of calomel with one of antimonial powder every three hours. In the evening the mercurial frictions were repeated on the arms. 18th. Continuation of the same means—antimoniated ptisan for drink.

19th. A remission of the symptoms—cessation of the convulsive movements—inability to move the left arm and leg, which have, however, preserved their temperature. Diarrhœa, at first watery, afterwards green and feculent. 21st. The favourable change continues, and the fever abates—the urine lets fall a copious sediment—the stools are abundant and fæcal—some tranquil sleep—an abscess forming at the angle of the lower jaw. Still the patient complains greatly of her head, and feels some ease by the pressure of the hand on the forehead. On the 4th February there was a discharge of purulent matter from both ears; and from this time the cephalalgia gradually abated, and finally ceased in March, the discharge from the ears continuing till the month of May. Mercury, in small quantities, was continued till the 15th March, the child having taken about 37 grains altogether. The convalescence was very tedious, the bowels remaining long torpid, and requiring lavements—the digestion impaired—and the recovery of strength very slow. The extract of cinchona, with a little malaga wine, conduced much to the restoration of strength. She is now stouter than any of her sisters.

With all due deference to Dr. Fallot, we do not consider the above case as any other than one of cerebral inflammation—most probably arachnitis, occasioned by cold. We know that all the phenomena exhibited above, even to the strabismus, convulsions, and dilated pupil, may be produced by inflammation of the cerebral investments, without any effusion. The purulent discharge from the ears too, corroborates our observations. We believe, however, that the case would have terminated in effusion, had not Dr. F. taken the measures detailed above, and which were, generally speaking, judicious, with the exception of the neglect of purging—a neglect of which the continental physicians are constantly guilty, in acute diseases.

